# Brazil's restoration blueprint for biodiversity credits

**DOI:** 10.1111/cobi.70063

**Published:** 2025-05-31

**Authors:** Maria Lucia Ferreira Barbosa, Carlos Delano Cardoso de Oliveira, Vinicius Tonetti, José Matheus Segre Moneva Viveiros, Gerd Sparovek, Jean Ometto, Paulo Guilherme Molin

**Affiliations:** ^1^ Centro de Ciências Natureza Universidade Federal de São Carlos Buri Brazil; ^2^ UK Centre for Ecology and Hydrology Wallingford UK; ^3^ Instituto de Estudos Avançados Universidade de São Paulo São Paulo Brazil; ^4^ Instituto Nacional de Pesquisas Espaciais (INPE) São José dos Campos Brazil; ^5^ Center for Carbon Research in Tropical Agriculture University of São Paulo Piracicaba Brazil; ^6^ Escola Superior de Agricultura Luiz de Queiroz da Universidade de São Paulo – USP Piracicaba SP Brazil

**Keywords:** Forest Restoration, Biodiversity credits, Carbon Market

The biodiversity credits market is a financial mechanism aimed at incentivizing the conservation and recovery of biological diversity by linking ecological outcomes to economic value. Evolving over 2 decades through diverse approaches (World Economic Forum, 2022), this market has gained prominence as a major global economic opportunity (Yan et al., 2024). At COP16 in 2024, new platforms were introduced to bolster the market's growth. An advisory panel led by the United Kingdom and France presented a framework for trading “high‐integrity” credits (IAPB, 2024), and Verra launched a system for creating nature credits (https://verra.org/verra‐launches‐nature‐framework/). In Brazil, some initiatives, such as the one led by Ecosystem Regeneration Associates (ERA), are already rewarding land stewards for preserving biodiversity, with a focus on conserving the jaguar (*Panthera onca*) as an umbrella species (https://www.erabrazil.com/biodiversity). Despite that, a targeted biodiversity credits market for Brazil is yet to be developed.

Brazil is the most biodiverse country in the world, boasting 6 terrestrial biomes, an extensive coastline, and 2 biodiversity hotspots (Myers et al., 2000). Yet, its biodiversity's potential to contribute to sustainable bioeconomic growth and development remains largely overlooked (Ellwanger et al., 2023). Although Brazil is uniquely positioned to lead in integrating biodiversity into its economic, environmental, and social policies, this integration requires a fundamental shift in how natural capital is valued and integrated in market‐based conservation strategies.

Brazil has demonstrated leadership in environmental conservation, notably during the 1992 Rio Earth Summit and by reducing Amazon deforestation by over 80% from 2004 to 2012. However, biodiversity loss remains a critical problem. For instance, forest fragments in Brazil's Atlantic Forest lost 25–32% of their biomass and 23–31% of their species diversity from 1985 to 2017 (Lima et al., 2020). Moreover, 65% of all tree species and 82% of endemic tree species in the Atlantic Forest are threatened (Lima et al., 2024). Tree losses directly reduce biomass and, consequently, carbon storage capacity. In the Atlantic Forest, this decline has led to an estimated US$2.3–2.6 billion in lost carbon credits (Lima et al., 2020). Still, carbon credits fail to capture the broader ecological decline, including reduced ecosystem resilience.

Given the accelerating global loss of biodiversity and climate change, restoring ecosystems has never been more pressing. Ecological restoration in Brazil has advanced significantly (Rodrigues et al., 2009; Rother et al., 2023) because it is underpinned by robust legal frameworks to reverse ecosystem degradation (Brazil, 2012; 2017). Supported by a national ecological restoration society (https://sobrestauracao.org/) and regional and biome‐level organizations (e.g., https://www.pactomataatlantica.org.br/), Brazil has exemplary restoration governance. These elements have driven ecological restoration initiatives across the country's 6 biomes (Guerra et al., 2020). For example, restoration efforts in the Atlantic Forest have been recognized as one of the 10 World Restoration Flagships of the UN Decade on Ecosystem Restoration (United Nations, 2025). Many areas of the Atlantic Forest are undergoing both natural regeneration and active restoration, with over 673,000 hectares of forest recovered between 2011 and 2015 (Crouzeilles et al., 2019).

The biodiversity market offers a novel mechanism to mobilize financial resources for restoration and conservation to ensure that ecosystem restoration projects focus not only on sequestering carbon but also on regaining ecosystem services and biodiversity. Restoration actively enhances biodiversity through the rehabilitation of degraded ecosystems, leading to measurable biodiversity gains, such as increased species richness and improved ecosystem function. In contrast, conservation focuses on preserving existing biodiversity levels, which, although essential, do not lead to the same additional biodiversity gains as restoration.

Biodiversity loss occurs through habitat destruction and gradual degradation processes, such as wildfires, species invasion, and climate change. Frameworks for biodiversity credits must consider degradation and outright habitat loss and consider the surrounding landscape and its ecological network to ensure holistic and context‐sensitive conservation. These systems should align with existing environmental policies and recognize biodiversity as essential to the long‐term success of initiatives, such as forest carbon projects.

Brazil's experience with carbon markets (Vargas et al., 2021) offers valuable lessons for shaping biodiversity credits. These markets are interconnected because forest carbon projects often contribute to biodiversity conservation, offering opportunities for cobenefits and integrated environmental solutions. Yet, biodiversity involves more intricate dynamics, such as species diversity, habitat integrity, and ecological processes, that require a sophisticated framework for quantification and valuation. A universal, one‐size‐fits‐all metric for biodiversity is neither practical nor desirable. However, the lack of reference data for original biodiversity in many areas further complicates the establishment of a credible system. Although technologies, such as environmental DNA and artificial intelligence, offer promising avenues for monitoring and assessing biodiversity (Allard et al., 2023; Ullah et al., 2025), they will not be viable on a large scale until they become affordable, accessible, and taxonomically inclusive (Stephenson, 2020).

To succeed, biodiversity markets must ensure that local communities—especially Indigenous peoples and small farmers—are involved and benefit from these initiatives. The Colombian Amazon offers an example of a successful program in which biodiversity credits were developed by and for Indigenous people before being adapted to the global market (https://pt‐br.savimbo.com/biodiversity). This demonstrates that biodiversity conservation can be equitable and impactful.

At COP16 (2024), Brazil's Indigenous peoples launched the manifesto “We Are the Answer” (APIB, 2024), calling for concrete actions to address the climate and biodiversity crises. They demanded active participation in COP30, which will be hosted in Brazil. Although COP16 released several new guidelines for biodiversity credits, progress on biodiversity financing remained limited, and Brazil failed to present its National Biodiversity Strategies and Action Plan. This year, COP30 offers Brazil a renewed opportunity to elevate biodiversity in global discussions by integrating biodiversity conservation with climate change mitigation. Such an integrated approach can drive more effective solutions because functioning ecosystems are essential for addressing climate change impacts.

The Atlantic Forest—one of the most endangered ecosystems in the world (Myers et al., 2000)—could serve as a flagship of how biodiversity credits can promote both conservation and economic development. By creating robust, science‐driven frameworks for biodiversity credits that account for ecological complexity and prioritize social equity, Brazil can lead the world in building a more resilient future.
